# Supplementary material for: Extensive variation and strain-specificity in dengue virus susceptibility among African Aedes aegypti populations
Source: bioRxiv. 2023 Dec 14:2023.12.14.571617. Preprint. [Version 1] doi: 10.1101/2023.12.14.571617 (PMC10760182; doi:10.1101/2023.12.14.571617)
Supplement: 1 [file NIHPP2023.12.14.571617v1-supplement-1.pdf]

bioRxiv preprint doi: https://doi.org/10.1101/2023.12.14.571617; this version posted December 14, 2023. The copyright holder for this preprint (which was not certified by peer review) is the author/funder, who has granted bioRxiv a license to display the preprint in perpetuity. It is made available under aCC-BY 4.0 International license.

Supplementary Figure

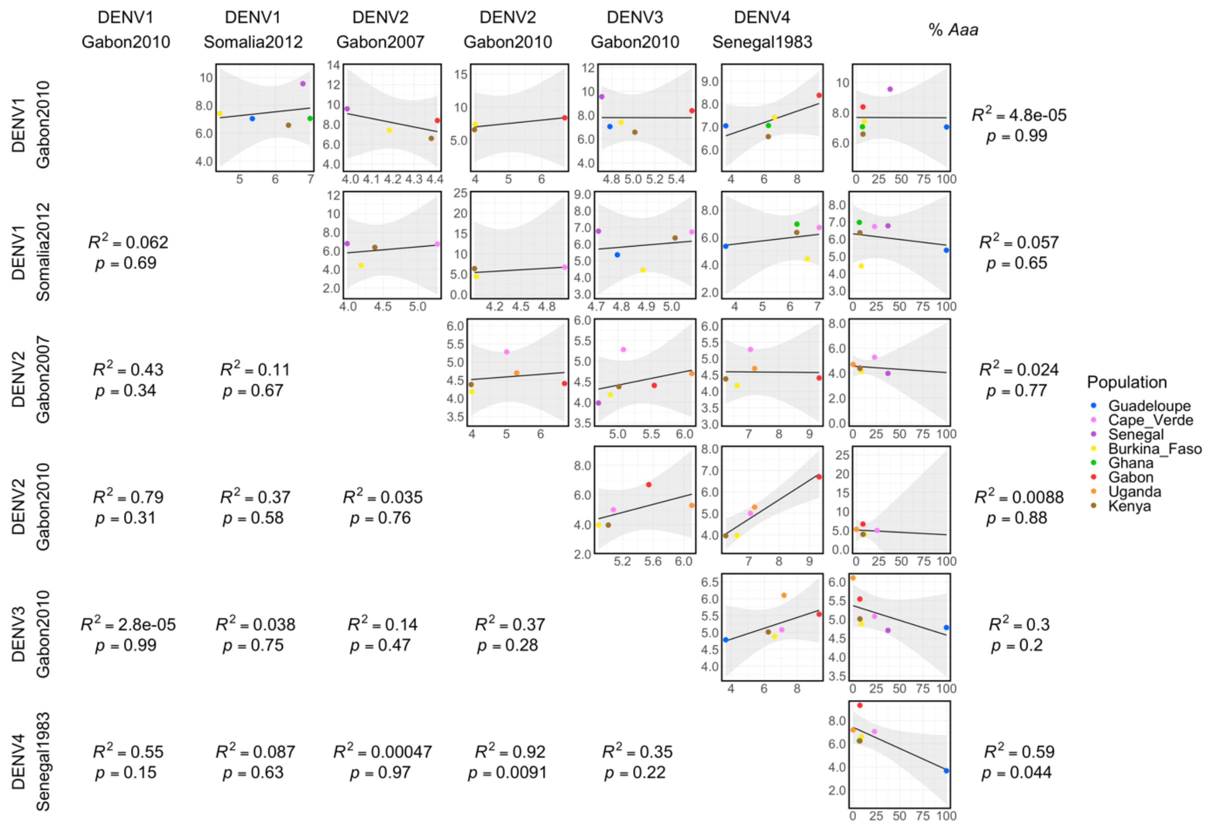

Figure S1. Correlations between DENV susceptibility levels for different virus strains and the percentage of *Aaa* ancestry. The Pearson linear correlations between OID_{50} estimates are shown for each pair of DENV strains and with % *Aaa* (rightmost column). The black lines represent the linear correlations, and the grey shading indicates their confidence interval. The mosquito populations are color-coded; their average % *Aaa* was determined based on whole-genome sequencing of their wild-caught progenitors.
